# On prognostic estimates of radiation risk in medicine and radiation protection

**DOI:** 10.1007/s00411-019-00794-1

**Published:** 2019-04-20

**Authors:** Alexander Ulanowski, Jan Christian Kaiser, Uwe Schneider, Linda Walsh

**Affiliations:** 1 Institute of Radiation Medicine, Helmholtz Zentrum München, German Research Center for Environmental Health, Ingolstädter Landstraße 1, 85764 Neuherberg, Germany; 2 International Atomic Energy Agency, IAEA Environmental Laboratories, 2444 Seibersdorf, Austria; 3 Department of Physics, Science Faculty, University of Zürich, Winterthurerstrasse 190, 8057 Zurich, Switzerland; 4 Radiotherapy Hirslanden, Witellikerstrasse 40, 8032 Zurich, Switzerland

**Keywords:** Radiation exposure, Risk assessment, Lifetime risk, Cumulative risk, Risk projections, Radiation protection, Medical use of radiation

## Abstract

The problem of expressing cumulative detrimental effect of radiation exposure is revisited. All conventionally used and computationally complex lifetime or time-integrated risks are based on current population and health statistical data, with unknown future secular trends, that are projected far into the future. It is shown that application of conventionally used lifetime or time-integrated attributable risks (LAR, AR) should be limited to exposures under 1 Gy. More general quantities, such as excess lifetime risk (ELR) and, to a lesser extent, risk of exposure-induced death (REID), are free of dose constraints, but are even more computationally complex than LAR and AR and rely on the unknown total radiation effect on demographic and health statistical data. Appropriate assessment of time-integrated risk of a specific outcome following high-dose (more than 1 Gy) exposure requires consideration of competing risks for other radiation-attributed outcomes and the resulting ELR estimate has an essentially non-linear dose response. Limitations caused by basing conventionally applied time-integrated risks on current population and health statistical data are that they are: (a) not well suited for risk estimates for atypical groups of exposed persons not readily represented by the general population; and (b) not optimal for risk projections decades into the future due to large uncertainties in developments of the future secular trends in the population-specific disease rates. Alternative disease-specific quantities, baseline and attributable survival fractions, based on reduction of survival chances are considered here and are shown to be very useful in circumventing most aspects of these limitations. Another main quantity, named as radiation-attributed decrease of survival (RADS), is recommended here to represent cumulative radiation risk conditional on survival until a certain age. RADS, historically known in statistical literature as “cumulative risk”, is only based on the radiation-attributed hazard and is insensitive to competing risks. Therefore, RADS is eminently suitable for risk projections in emergency situations and for estimating radiation risks for persons exposed after therapeutic or interventional medical applications of radiation or in other highly atypical groups of exposed persons, such as astronauts.

## Introduction

In recent years, there has been a rapid increase in the doses received from medical exposures to ionising radiation, in the US, Europe and other countries, which has fuelled concern about the long-term consequences of such exposures, particularly in terms of cancer induction (Hall and Brenner 2008). Medical exposures comprise applications of ionising radiation in radiology, nuclear medicine and radiotherapy. An estimate of the long-term consequences of these exposures can be particularly important for groups of exposed patients. Such groups include: radiology paediatric patients (Brenner et al. 2001); and radiotherapy patients treated for a primary malignancy related to high cure rates, or patients treated for benign disease (Newhauser and Durante 2011; Schneider 2011). In determining the choice of treatment for radiotherapy patients with benign disease, a knowledge of the subsequent lifetime cancer risks needs to be taken into consideration and may have ethical implications which also need to be considered, for example in the treatment of trauma patients (Oertel et al. 2008). When new treatment/imaging techniques or radiation qualities are clinically introduced, it is also important to assess the potential long-term consequences, in terms of the associated lifetime cancer risk.

The concerns about the long-term consequences of radiation exposures are not only restricted to medically exposed patients, but also include persons exposed after emergency situations (such as nuclear accidents) and occupationally exposed persons. Past health risk assessments after nuclear accidents, such as in Fukushima in 2011 (WHO 2013; Walsh et al. 2014), have utilized the conventional lifetime risk measure of lifetime attributable risk (LAR), that involves projecting current cancer rates and mortality rates far into the future. The unsatisfactory nature of such an assumption, that current population rates will remain constant far into the future, has already been noted in detail (Walsh et al. 2014). For astronauts, the health risks from radiation exposures accumulated during missions can become one of the major risk factors, when considering bases on the Moon, or missions to Mars.

The aforementioned groups of medically and occupationally exposed persons are highly atypical, in the sense that they do not represent the general population in terms of baseline cancer risks. As a consequence, baseline rates and survival functions pertaining to the general population are poor approximations to use in assessing the radiation related cancer risks pertaining to these specific groups. Unfortunately, all currently used predictors of lifetime cancer risk of radiation exposures are based on estimates of such baseline rates and/or survival functions.

The goal of this paper is to establish the usefulness, through a novel application in radiation risk assessment, of a quantity that is eminently suitable for estimating time-integrated or lifetime risks for exposed groups of persons. This quantity, historically known in statistical literature as “cumulative risk” and named here as radiation-attributed decrease of survival (RADS), represent cumulative radiation risk conditional on survival until a certain age. RADS is only based on the radiation-attributed hazard and is insensitive to competing risks. It is shown here that the methodology behind the novel application of RADS to radiation risk assessment confers a suitability for applications to exposed groups that are not represented by the broad attributes of the general population. A further suitability comes from not requiring the assumption, that current population rates will remain constant far into the future because this novel application is independent of current trends and unknown future secular trends in population survival functions.

## Available quantities for lifetime radiation risk estimates

Lifetime attributable risk is a widely adopted and commonly used measure of integral harmful effects of radiation exposure (Vaeth and Pierce 1990; Thomas et al. 1992; UNSCEAR 1994, 2000; Kellerer et al. 2001; BEIR 2006; EPA 2011; WHO 2013; Walsh et al. 2014). As introduced by Vaeth and Pierce (1990), an integration of failure rates (here, a mortality rate^1^), taking into account conditional survival probability and assuming negligible effect of radiation exposure on the general survival, leads to the following equations for calculating lifetime baseline risk (LBR) of death due to a specific cause *c*, where the dependence on gender is omitted for brevity and notations suggested by Thomas et al. (1992) are used:

1 $${\text{LBR}}_{c} (e) = \frac{1}{S(e)}\mathop \int \limits_{e}^{\infty } \mu_{c} (u)S(u) {\text{d}}u,$$

and, correspondingly, lifetime radiation-attributable risk:

2 $${\text{LAR}}_{c} (e,D) = \frac{1}{S(e)}\mathop \int \limits_{e}^{\infty } h_{c} \left( {u |e,D} \right) S(u){\text{d}}u,$$

where $\mu_{c} (u)$ is the baseline mortality rate^2^ due to the cause *c* (PY^−1^); $h_{c} (u|e,D)$ is the excess mortality rate due to the cause *c* attributable to radiation exposure with dose *D* at age *e* (PY^−1^) and, if cause *c* is cancer, the latent period before any possible development is accounted for; *e* and *a* are the age at exposure and the attained age (year), respectively; $S(u)$ is the survival function for an unexposed population (dimensionless, see “Appendix”). The Eqs. (1) and (2) can be interpreted as time-integrated baseline and excess mortality rates accounting for age-dependent survival $S(e)$ for subjects being alive and exposed to radiation at age *e*. The resulting integral quantities, LAR and LBR, are proportions and can be further interpreted as probabilities (odds) to ‘fail’, i.e., representing chances to die either due to preceding radiation exposure (LAR) or spontaneously due to other, non-radiation attributable, causes (LBR) during the full lifetime following the radiation exposure at age *e*. Excess rates $h_{c}$ are inferred from pertinent models of radiation risk, thus $h_{c} = {\text{EAR}}_{c}$ for an excess absolute risk (EAR-type) model or $h_{c} = {\text{ERR}}_{c} \mu_{c}$ for an excess relative risk (ERR-type) model.^3^

If the upper integration limit is set to be less than the lifetime, then Eqs. (1) and (2) turn into definitions of time-integrated baseline and attributable risks:

3 $${\text{BR}}_{c} \left( {a|e} \right) = \frac{1}{S(e)}\mathop \int \limits_{e}^{a} \mu_{c} (u)S(u){\text{d}}u,$$

4 $${\text{AR}}_{c} \left( {a|e,D} \right) = \frac{1}{S(e)}\mathop \int \limits_{e}^{a} h_{c} \left( {u |e,D} \right)S(u){\text{d}}u.$$

Distinctive features of Eqs. (2) and (4) are implicit assumptions that the impact of radiation on the general survival function is negligible and that future radiation-attributed risk can be derived using contemporary survival and health statistics data for the general (unexposed) population.

Other widely considered definitions of time-integrated radiation risk also include excess lifetime risk (ELR) and risk of exposure-induced death (REID) or risk of exposure-induced cancer (REIC) (see e.g. Vaeth and Pierce 1990; Thomas et al. 1992; Kellerer et al. 2001; UNSCEAR 1994), which differ from the equations above by accounting for an effect of radiation exposure on general survival after exposure.

The former quantity, ELR, is defined as follows:

5 $${\text{ELR}}_{c} (e,D) = \frac{1}{S(e)}\left[ {\mathop \int \limits_{e}^{\infty } \mu_{c}^{*} \left( {u |e,D} \right) S^{*} \left( {u |e,D} \right) {\text{d}}u - \mathop \int \limits_{e}^{\infty } \mu_{c} (u) S(u) {\text{d}}u } \right],$$

where $\mu_{c}^{*} ( \cdot )$ and $S^{*} ( \cdot )$ are the cause *c* mortality rate and the survival function for the exposed population, correspondingly. ELR is the most general representation of the lifetime radiation-attributed risk, which adequately takes into account effects of competing radiation-attributable risks on the survival function. Similarly, to Eq. (4), the excess risk (ER) can be defined as follows:

6 $${\text{ER}}_{c} \left( {a |e,D} \right) = \frac{1}{S(e)}\left[ {\mathop \int \limits_{e}^{a} \mu_{c}^{*} \left( {u |e,D} \right) S^{*} \left( {u |e,D} \right) {\text{d}}u - \mathop \int \limits_{e}^{a} \mu_{c} (u)S(u){\text{d}}u } \right].$$

Another quantity, REID (for mortality rates) or REIC (for incidence rates), neglects the difference between radiation-affected and general survival functions and integrates the risk as follows:

7 $${\text{REID}}_{c} \left( {a |e,D} \right) = \frac{1}{S(e)}\mathop \int \limits_{e}^{a} \left( {\mu_{c}^{*} \left( {u |e,D} \right) - \mu_{c} (u)} \right) S^{*} \left( {u |e,D} \right){\text{d}}u.$$

It was shown (Kellerer et al. 2001), and is also demonstrated in the “Appendix”, that these quantities converge to Eqs. (2) and (4) for dose ranges under 1 Gy, thus clearly demonstrating that LAR (Eq. 2) and AR (Eq. 4) have applicability domain restrictions.

To compute radiation risk estimates using the conventional quantities, LAR/AR, LBR/BR, ELR, and REID/REIC, one needs to know not only radiation-attributed mortality/incidence rates but also the survival functions as well as the baseline rates for the outcomes of interest. This can be a task fraught with difficulties in situations where contemporary population and health statistics are not representative for a population or an individual of interest. For example, risk projections for general population groups affected by accidental radiation exposures, when risks of radiation effects must be assessed in an emergency to support decision making on protective measures and actions. Another situation challenging the conventional quantities arises from medical radiation exposures, diagnostic and therapeutic, since baseline risks and survival chances for patients are not always representative of those for the general, mostly healthy, population. An even more challenging situation appears when radiation is applied to treat cancer. Individual survival chances for cancer patients strongly depend on the diagnosed stage, and on other individual properties, thus making risk projections using life and health statistics for the general population highly uncertain and approximate. Radiotherapy also affects individual survival chances for the cancer patients making the risk estimates even more uncertain. Realisation of these difficulties in the risk projections for the above-mentioned situations, provided a motivation basis for the present work.

## Estimation of the future radiation risk

### Time-integrated excess risk

The conventional equations for attributable and spontaneous risks (Eqs. 1–7) represent estimates of cumulative risks by time-integrating the relative number of fatalities (cases), which is expressed as a product of hazard and the survival function (see “Appendix”, Eq. 16). Due to this, for computation of the conventional quantities, data for representative population are required: model-based hazard rates, baseline rates, and survival functions from age at exposure to lifespan or to a specific age.

In the following, and similarly to earlier works (Vaeth and Pierce 1990; Thomas et al. 1992), consider two identical populations of the same sex and the same age *e*: unexposed and exposed to radiation. The unexposed population can be characterised by the spontaneous all-cause $\mu (t)$ and cause-specific $\mu_{i} (t)$ mortality rates and the general survival function $S(t |e)$ conditional on survival until age *e*. Similarly, the exposed population can be characterised by excess mortality rates, all-cause $h(t)$ and cause-specific $h_{i} (t)$, and by the total (baseline and excess) mortality rates, $\mu^{*} (t) = \mu (t) + h(t)$ and $\mu_{i}^{*} (t) = \mu_{i} (t) + h_{i} (t)$. The full set of quantities and terms used in the current paper is shown in Table 1, where all quantities are defined, and the notation is summarised. The use of the notation serves to abbreviate and to simplify equations given below in the following text. For example, explicit indications of the fact that all survival functions and cumulative mortality rates are conditional on survival to the age at exposure *e*, are generally omitted in the notation.

**Table 1:** Definition of terms and notations used

Mortality type	Cause	Mortality rates		Cumulative mortality rates		Survival	
		Definition	Notation	Definition	Notation	Definition	Notation
Baseline	*i*	$\mu_{i} (t)$		$M_{i} (t |e) = \int_{e}^{t} {\mu_{i} (u ) {\text{d}}u}$	$M_{i} (t)$	$S_{i} (t |e) = \exp ( - M_{i} (t|e))$	$S_{i} (t)$
	All	$\mu (t) = \sum\nolimits_{i} {\mu_{i} (t)}$		$M(t |e) = \int_{e}^{t} {\mu (u){\text{d}}u}$	$M(t)$	$S(t |e) = \exp ( - M(t|e))$	$S(t)$
	All but *c*	$\mu_{d} (t) = \sum\nolimits_{i \ne c} {\mu_{i} (t)}$		$M_{d} (t |e) = \int_{e}^{t} {\mu_{d} (u ) {\text{d}}u}$	$M_{d} (t)$	$S_{d} (t |e) = \exp ( - M_{d} (t|e))$	$S_{d} (t)$
Excess (radiation-attributable)	*i*	$h_{i} (t |e,D)$	$h_{i} (t)$	$H_{i} (t |e,D) = \int_{e}^{t} {h_{i} (u|e,D ) {\text{d}}u}$	$H_{i} (t)$		
	All	$h(t|e,D) = \sum\nolimits_{i} {h_{i} (t|e,D)}$	$h(t)$	$H(t |e,D) = \int_{e}^{t} {h(u|e,D){\text{d}}u}$	$H(t)$		
	All but *c*	$h_{d} (t |e,D) = \sum\nolimits_{i \ne c} {h_{i} (t|e,D)}$	$h_{d} (t)$	$H_{d} (t |e,D) = \int_{e}^{t} {h_{d} (u |e,D ) {\text{d}}u}$	$H_{d} (t)$		
Total (in exposed population)	*i*	$\mu_{i}^{*} (t|e,D)$	$\mu_{i}^{*} (t)$	$M_{i}^{*} (t |e) = \int_{e}^{t} {\mu_{i}^{*} (u|e,D ) {\text{d}}u}$	$M_{i}^{*} (t)$	$S_{i}^{*} (t |e,D) = \exp ( - M_{i} (t |e) - H_{i} (t|e,D))$	$S_{i}^{*} (t)$
	All	$\mu^{*} (t |e,D) = \sum\nolimits_{i} {\mu_{i}^{*} (t|e,D)}$	$\mu^{*} (t)$	$M^{*} (t |e) = \int_{e}^{t} {\mu^{*} (u|e,D ) {\text{d}}u}$	$M^{*} (t)$	$S^{*} (t |e,D) = \exp ( - M(t |e) - H(t|e,D))$	$S^{*} (t)$
	All but *c*	$\mu_{d}^{*} (t |e,D) = \sum\nolimits_{i \ne c} {\mu_{i}^{*} (t|e,D)}$	$\mu_{d}^{*} (t)$	$M_{d}^{*} (t |e) = \int_{e}^{t} {\mu^{*} (u|e,D ) {\text{d}}u}$	$M_{d}^{*} (t)$	$S_{d}^{*} (t |e,D) = \exp ( - M_{d}^{*} (t |e) - H_{d}^{*} (t|e,D))$	$S_{d}^{*} (t)$

For brevity, assume that radiation exposure affects mortality rates for the cause *c*, only. This assumption is not restrictive, because the considered cause *c* may represent not a single cause but a composite outcome including several mortality causes affected by the radiation exposure. Therefore, in the following text, only the cause *c* will be considered as affected by radiation exposure. Consideration of the general situation, when only a single specific cause is of interest and radiation affects hazard rates for other causes, can be found in the “Appendix”.

For the single radiation-affected cause *c*, the conditional survival function of the exposed population can be represented as a product of the general survival and of the factor representing the radiation effect (see “Appendix” for details):

8 $$S^{*} \left( {t|e} \right) = \exp \left( { - \mathop \int \limits_{e}^{t} \mu_{c}^{*} \left( {u |e,D} \right){\text{d}}u} \right) = \exp \left( { - \mathop \int \limits_{e}^{t} \left[ {h_{c} (u|e,D) + \mu (u)} \right] {\text{d}}u} \right) = \exp \left( { - H_{c} \left( {t |e,D} \right)} \right)S(t|e),$$

or, using the abbreviated notation (see Table 1), as:

9 $$S^{*} (t) = \exp \left( { - \mathop \int \limits_{e}^{t} \mu_{c}^{*} (u){\text{d}}u} \right) = \exp \left( { - \mathop \int \limits_{e}^{t} \left[ {h_{c} (u) + \mu (u)} \right] {\text{d}}u} \right) = \exp \left( { - H_{c} (t)} \right)S(t).$$

**Fig. 1:**
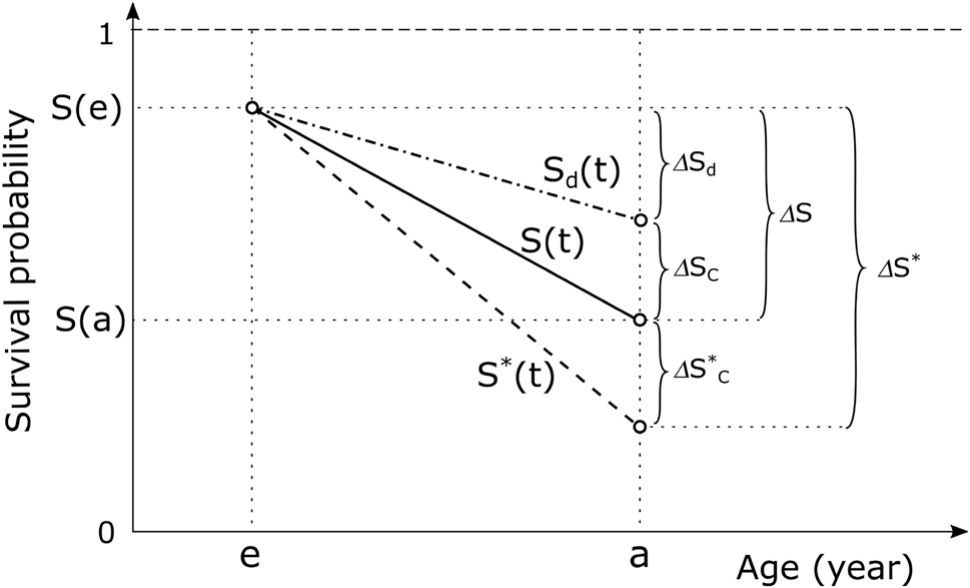
Illustration for the definitions of radiation risk, baseline and attributable fractions. $S(t)$ is the all-cause survival for unexposed population (solid line), $S_{d} (t)$ is the all-cause-but-cause-*c* survival for unexposed population (dash-dot line), $S^{*} (t)$ is the all-cause survival for exposed population (dash line)

Here, the detrimental effect of radiation exposure results in a reduction of survival chances in the exposed population $S^{*} (t)$ compared to that in the unexposed $S(t)$ by the factor $\exp ( - H_{c} (t))$. The situation is illustrated in Fig. 1, where survival curves for unexposed and exposed populations are shown schematically as well as the curve indicating the chances to survive all other mortality causes but *c*, $S_{d} (t)$. Also shown in the figure are fractions of population alive at age *e* which will be lost until age *a* due to: (a) all causes in exposed population, $\Delta S^{*}$, (b) radiation-attributed cause *c* in the exposed population, $\Delta S_{c}^{*}$, (c) all spontaneous causes in the unexposed population, $\Delta S$, (d) spontaneous cause *c*, $\Delta S_{c}$, (e) all-but-cause-*c* spontaneous causes, $\Delta S_{d}$.

With factorisation of the survival function (Eq. 9) and applying notations from Table 1, the excess risk formula (Eq. 6) for a single radiation-attributed outcome *c* can be re-written as follows:

10 $${\text{ER}}_{c} (a) = \mathop \int \limits_{e}^{a} \mu_{c} (u)S(u)\left[ {\exp \left( { - H_{c} (u)} \right)\left( {1 + \frac{{h_{c} (u)}}{{\mu_{c} (x)}}} \right) - 1} \right]{\text{d}}u.$$

The above equation is similar to that introduced earlier for excess cancer deaths, ECD, (UNSCEAR 2006 Vol. I, Annex A, Appendix B, B2) but it is not limited to the excess relative risk model to represent radiation-attributed risk as suggested in the UNSCEAR 2006 Report. Apparently, the above Eq. (10) at low dose exposures, when cumulative hazard $H_{c} (u)$ is small, converges to the conventional definition of AR (Eq. 4). Another remarkable property of the excess risk (Eq. 10), which was also noted in (UNSCEAR 2006), is an inherent non-linear dose response of the time-integrated risk. Indeed, even for risk models leading to hazard with linear dependence on dose $h_{c} (u)\sim D$ the exponential of the cumulative hazard $H_{c} (u)$ in (Eq. 10) results in non-linear dose dependence of the time-integrated excess risk. Accounting for effects of competing radiation-attributed risks (see “Appendix”, Eq. 24) makes this non-linearity even stronger.

Another important effect of radiation-attributed competing risk is that the baseline (spontaneous) rate in the exposed population is less than that in the non-exposed one due to reduced survival chances and, correspondingly, appears as a function of dose (see “Appendix”, Eq. 25). This effect is responsible for apparent reduction of ER/ELR (Eqs. 6, 10) at older ages, which was previously pointed out and discussed by Thomas et al. (1992).

The time-integrated conventional (Eqs. 2, 4–7) and generalised (Eq. 10) formulas represent total failures within the period from exposure to a certain age or lifetime, thus they provide estimates of probability to fail within the considered period of time and answer the question: “What are the chances to die from a radiation-attributed cause during a period of time from exposure to a certain age or lifetime?”.

### Radiation-attributed changes of survival

Alternative quantities to express radiation risk can be suggested using the differences in survival functions for exposed and unexposed populations (see Eq. 9). The survival function $S(a)$ represents probability of lifetime to exceed age *a* or, in other words, probability to survive to age *a* (see “Appendix”). As shown in Fig. 1, excess hazard from radiation exposure reduces survival chances at age *a* by $\Delta S_{c}^{*} = S(a) - S^{*} (a)$. This means that the conditional reduction of survival or radiation-attributable fraction, ${\text{AF}}_{c} = \Delta S_{c}^{*} /S(e)$, can be represented^4^ as

11 $${\text{AF}}_{c} (a) = \Delta S_{c}^{*} = S(a) - S^{*} (a) = S(a) \left( {1 - \exp \left( { - H_{c} (a)} \right)} \right),$$

where only point values of the general survival at ages *e* and *a* and the cumulated radiation-attributable excess rate are involved. The attributable fraction (Eq. 11) represents reduced chances to survive to age *a* due to additional radiation-attributed hazard.

Similarly, the baseline fraction, representing survival reduction for non-exposed population due to mortality from spontaneous, not related to radiation, cause *c* within a period from *e* to *a* can be written as follows (see Fig. 1)

12 $${\text{BF}}_{c} (a) = \Delta S_{c} = S_{d} (a) - S(a) = S(a)(\exp (M_{c} (a)) - 1),$$

where, the following factorisation of survival function has been used:

13 $$S(t) = \exp ( - M_{c} (t))S_{d} (t),$$

and $S_{d} (t) = \prod\nolimits_{i \ne c} {\exp ( - M_{i} (t))}$ is the function describing survival from all causes but the cause *c* (see Fig. 1).

The fractions (Eqs. 11, 12) are expressed as shares of the population alive at age *e* and not surviving beyond age *a* due to mortality from radiation-attributed (Eq. 11) or spontaneous (Eq. 12) cause *c* and, due to this, computation of both fractions requires knowledge of population survival at given ages and estimation of the baseline fraction (Eq. 12) additionally involves integration of contemporary baseline mortality or incidence rates for the selected cause *c*. The attributable fraction (Eq. 11) under the name “crude radiation risk” was once suggested to express “additional risk from radiation exposure in the presence of all other competing risks” (Groer 1980) but did not find common use in radiation risk modelling.

Contemporary demographic and health statistics are not necessarily optimal for risk projections decades into the future due to large uncertainties in the unknown future developments of secular trends in such data. Therefore, risk projections based on contemporary statistics may be unpredictably biased by such unrealistic assumptions. This situation becomes even more complex when risk projections are to be made for medical applications of radiation, e.g., in the case of radiotherapy for cancer. Survival chances for the general population are not representative for the cancer patients, their survival chances also depend strongly on diagnosis and on the cancer stage at diagnosis. Additionally, medical radiation treatment affects the relative survival chances of the cancer patients, thus sometimes resulting in apparently paradoxical situations when the better treatment plan, which maximises the patient’s survival chances, is characterised by highest cumulated risk estimates (LAR or AR) for a second primary cancer than alternative treatment plans, which result in poorer survival chances.

To avoid uncertainties in risk projections due to unknown future changes of the population statistics or varying personal survival chances due to a disease and its medical treatment, a novel application of an alternative quantity is introduced here to describe the detrimental effect of radiation exposure at arbitrary times of life following the radiation exposure. Namely, effect of radiation-attributed deaths due to cause *c* at or before age *a* following exposure at age *e* can be expressed as a radiation-attributed decrease of survival (RADS) at age *a* (cf. Eq. 10):

14 $${\text{RADS}}_{c} \left( {a|e,D} \right) = \frac{{\Delta S_{c}^{*} }}{S(a)} = \frac{{S(a) - S^{*} (a)}}{S(a)} = 1 - \exp \left( { - H_{c} (a|e,D)} \right).$$

RADS represents the fraction of survival chances of unexposed population which would be lost due to detrimental effects of radiation exposure. RADS is historically known in statistical literature as cumulative risk, i.e. “a measure of cancer risk when there are no censored observations, that is, in the absence of mortality” (Esteve et al. 1994) and “… there are no other competing risks…” (Sasieni et al. 2011).

**Fig. 2:**
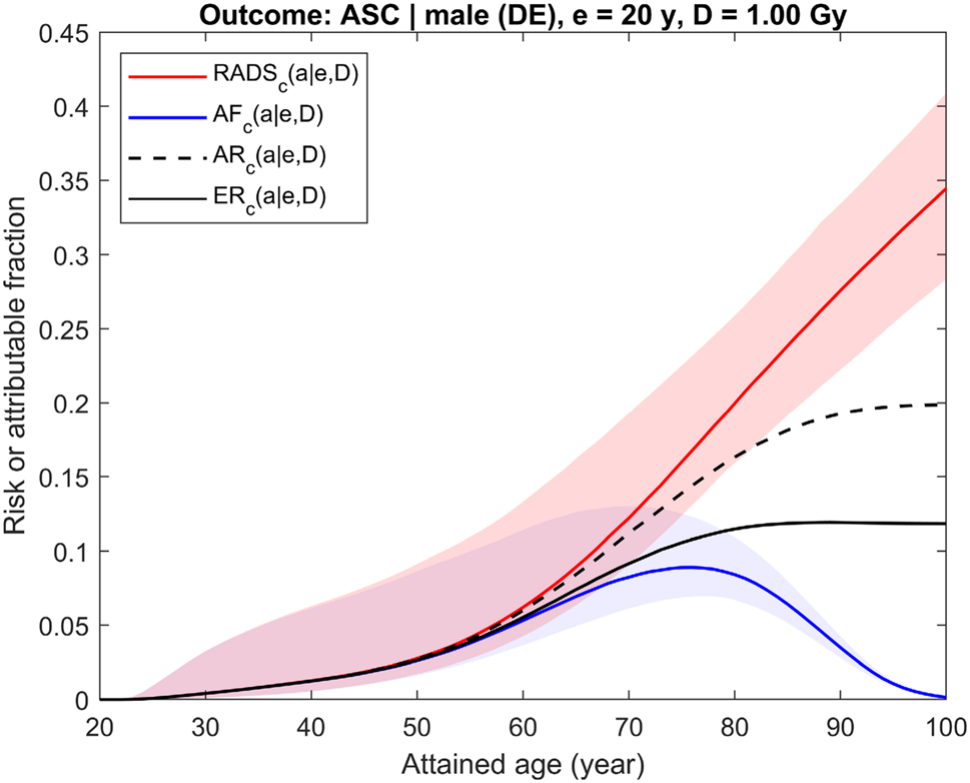
Illustration of differences between time-integrated risk estimates using conventional quantities AR/LAR (dashed black line), ER/ELR (solid black line) and the risk quantities suggested here AF and RADS (solid blue and red lines, respectively). Risks shown are for all solid cancers’ incidence for male from the contemporary (2013–2015) German population and exposed at age 20 years to 1 Gy colon dose using risk models as described in Walsh et al. (2019). The shaded areas represent 95% CI of estimates (colour figure online)

**Fig. 3:**
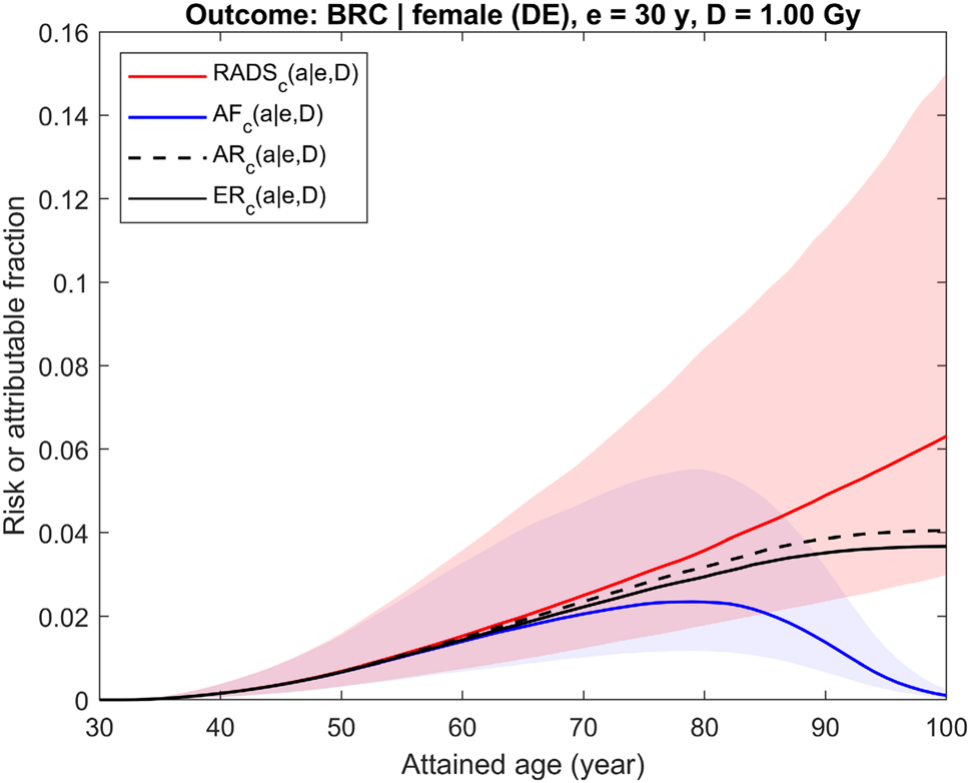
Illustration of differences between time-integrated risk estimates using conventional quantities AR/LAR (dashed black line), ER/ELR (solid black line) and the risk quantities suggested here AF and RADS (solid blue and red lines, respectively). Risks shown are for breast cancer incidence for female from the contemporary (2013–2015) German population exposed at age 30 years to 1 Gy breast dose using risk models as described in Walsh et al. (2019). The shaded areas represent 95% CI of estimates (colour figure online)

The differences between AR/LAR, ER/ELR, AF, and RADS are illustrated by Figs. 2 and 3, where risks and attributable fractions are plotted together. The plots represent the effects of radiation exposure at dose of 1 Gy on incidence of all solid cancers for a male (Fig. 2) and of female breast cancer (Fig. 3). Equations for mortality introduced above for all plotted quantities remain valid for disease incidence rates as well, provided the survival curves in the equations are redefined to represent chances to survive disease-free to a certain age (see more on this “Appendix”, Eqs. 26–28). The population data used for calculations are based on demographic and health statistics in Germany in 2013–2015 (Statistisches Bundesamt 2016; RKI-GEKID 2017) and the models of radiation risk are from the Life Span Study cohort in case of all solid cancers (Grant et al. 2017) and from the pooled cohort for female breast cancer (Preston et al. 2002). The risk calculation and uncertainty estimation techniques are the same as presented in Ulanowski et al. (2016) and described in Walsh et al. (2019).

Shown in Fig. 2 are prognostic estimates of radiation risk and the attributable fraction of an aggregated outcome represented by the occurrence of any solid cancer in a male exposed at age 20 years to a colon dose of 1 Gy. The next figure, Fig. 3, presents the same estimates made for radiation risk of a single disease, female breast cancer, following an exposure to 1 Gy breast dose at age 30 years. Both figures show that AR (Eq. 4), when compared to ER (Eq. 6), overestimates radiation risk because the effect of reduced survival following the exposure is neglected in AR. Attributable fraction AF (Eq. 11), representing chances to survive beyond a certain age, follows the behaviour of the population survival function and, at older ages and lower survival chances, gradually reduces to zero. RADS (Eq. 14), being free of competing risks effects, represents a detrimental effect of radiation exposure and shows the fraction of survival chances which will be lost at a certain age due to the exposure. An important property of all risk estimates is that their values are remarkably close to each other, despite the very different calculation methods, at ages less than 50–60 years, when survival chances are close to 1 and all risk estimates are not noticeably affected by the competing risks that are not attributed to radiation exposure.

Note that, unlike attributable and baseline fractions given in Eqs. (11) and (12), respectively, RADS (Eq. 14) is not a share of the population exposed at age *e* not surviving beyond a certain age. Instead, RADS is a factor modifying survival, so it represents the cumulated effect of radiation-attributed risk to ‘fail’ (to die or to become diseased) prior to or at the specified age *a*. It answers the question: “How much will the personal chances to survive to a certain age be reduced by detrimental effects of radiation?” As a factor modifying survival, it is meaningless for time beyond lifetime, where the survival is zero.

### Years of life lost

The integral of a survival function, i.e., the area below the survival curve, represents the number of person-years for the relevant population. Due to a normalisation of the survival function, this integral from 0 to infinity, is numerically equal to the mean lifetime in the population. Correspondingly, integration of the survival function for the exposed population will result in the reduced value of the mean lifetime: therefore, another important quantity, years of life lost (YLL) or loss of life expectancy (LLE, see e.g. Vaeth and Pierce 1990; UNSCEAR 1994), can be calculated using the general population survival function, $S(t)$, and the radiation-attributed (model) excess rate(s) as follows:

15 $${\text{YLL}}(e,D) = \Delta T\left( {e,D} \right) = \mathop \int \limits_{0}^{\infty } \left( {S(u) - S^{*} (u|e,D)} \right){\text{d}}u = \mathop \int \limits_{0}^{\infty } S(u)\left( {1 - \exp \left( { - H(u|e,D)} \right)} \right){\text{d}}u.$$

The formula (Eq. 15) is similar to one in the UNSCEAR 2006 Report (UNSCEAR 2006, Annex A, Appendix B, B4) but as in the case of excess risk (Eq. 10) is free from restricting assumptions on the type of radiation risk model.

## Sample calculations

In this section, the conventional risk quantities (Eqs. 4, 6, 7) and RADS (Eq. 14) are plotted together for several exemplary cases varying in disease outcome, gender, and dose for exposure at age 10 years. Risks of getting diseased with a solid or thyroid cancer are used in these exemplary cases, thus all risk computations are done for incidence of the respective diseases and the survival function is re-defined to represent disease-free survival, as described in “Appendix” and explained in the previous section for Figs. 2 and 3. More details on the cancer risk models and the computation technique used can be also found in Walsh et al. (2019).

**Fig. 4:**
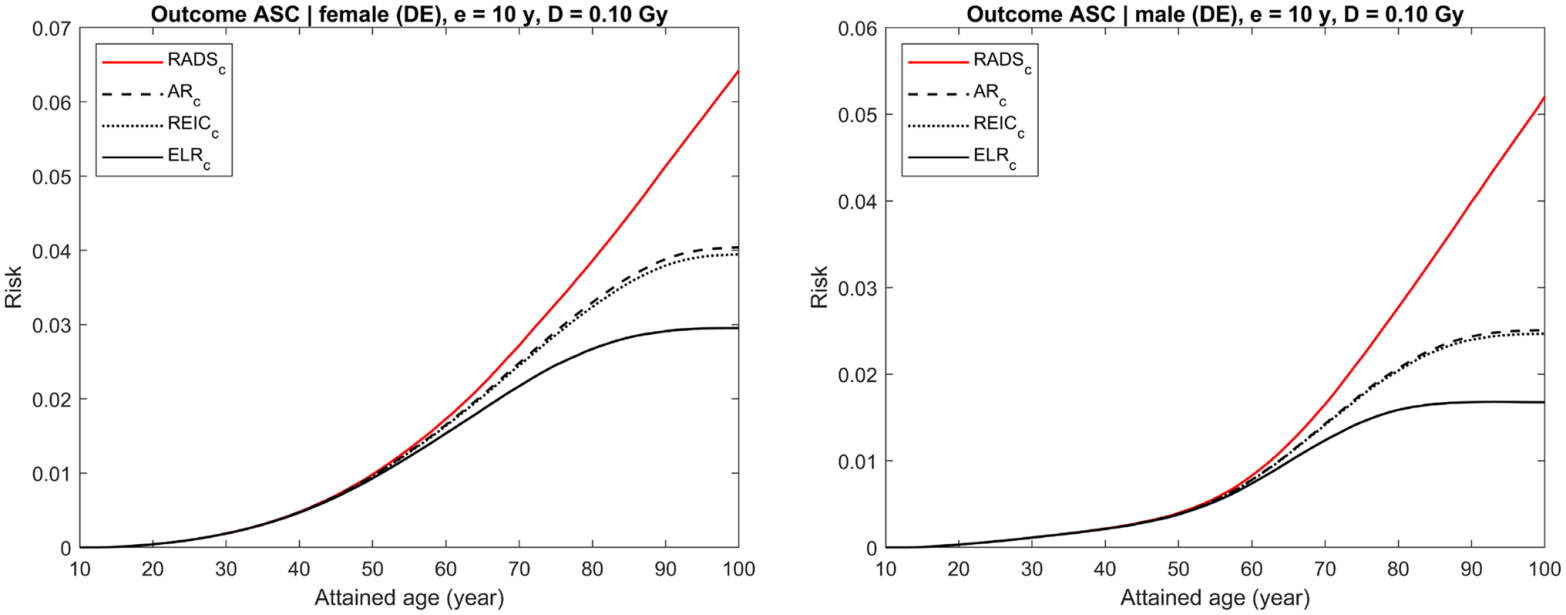
Radiation-attributed risks of all solid cancer incidence for female (left plot) and male (right plot) following exposure at age 10 years to 0.1 Gy colon dose computed using different risk quantities: AR (black dashed line), REIC (black dotted line), ELR (black solid line), and RADS (red solid line) (colour figure online)

**Fig. 5:**
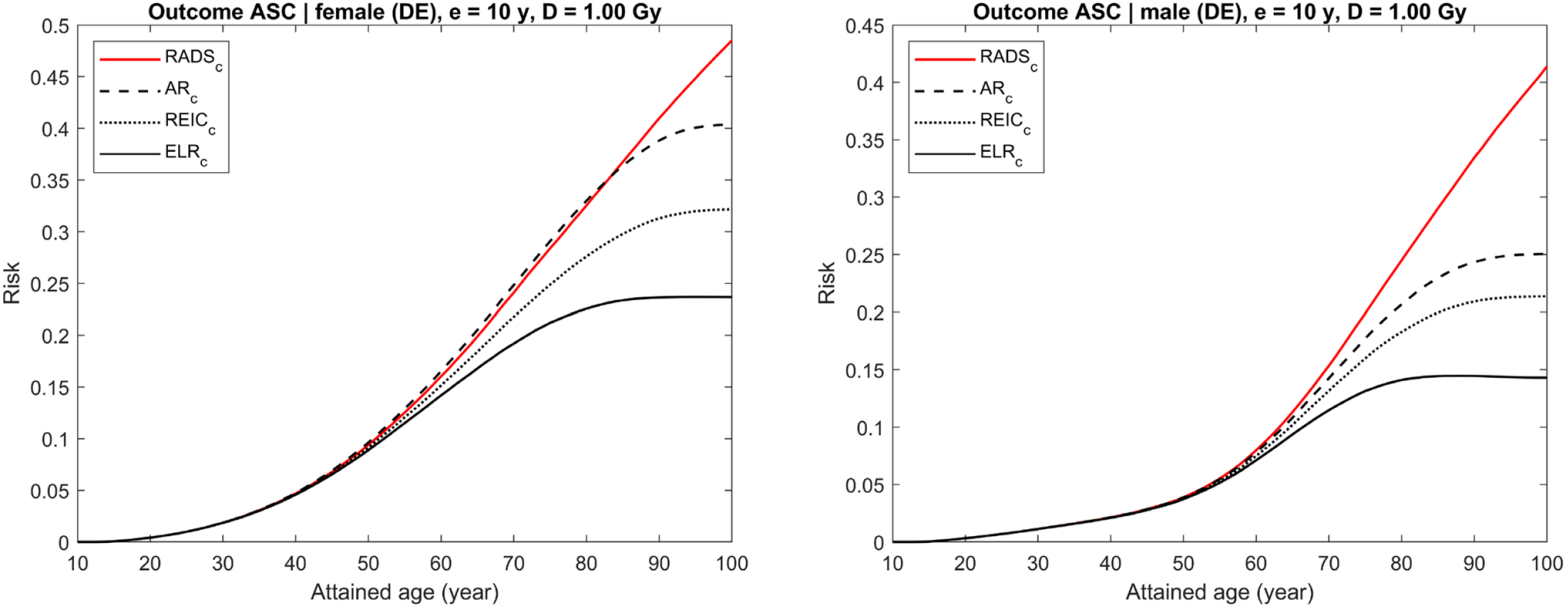
Radiation-attributed risks of all solid cancer incidence for female (left plot) and male (right plot) following exposure at age 10 years to 1 Gy colon dose computed using different risk quantities: AR (black dashed line), REIC (black dotted line), ELR (black solid line), and RADS (red solid line) (colour figure online)

**Fig. 6:**
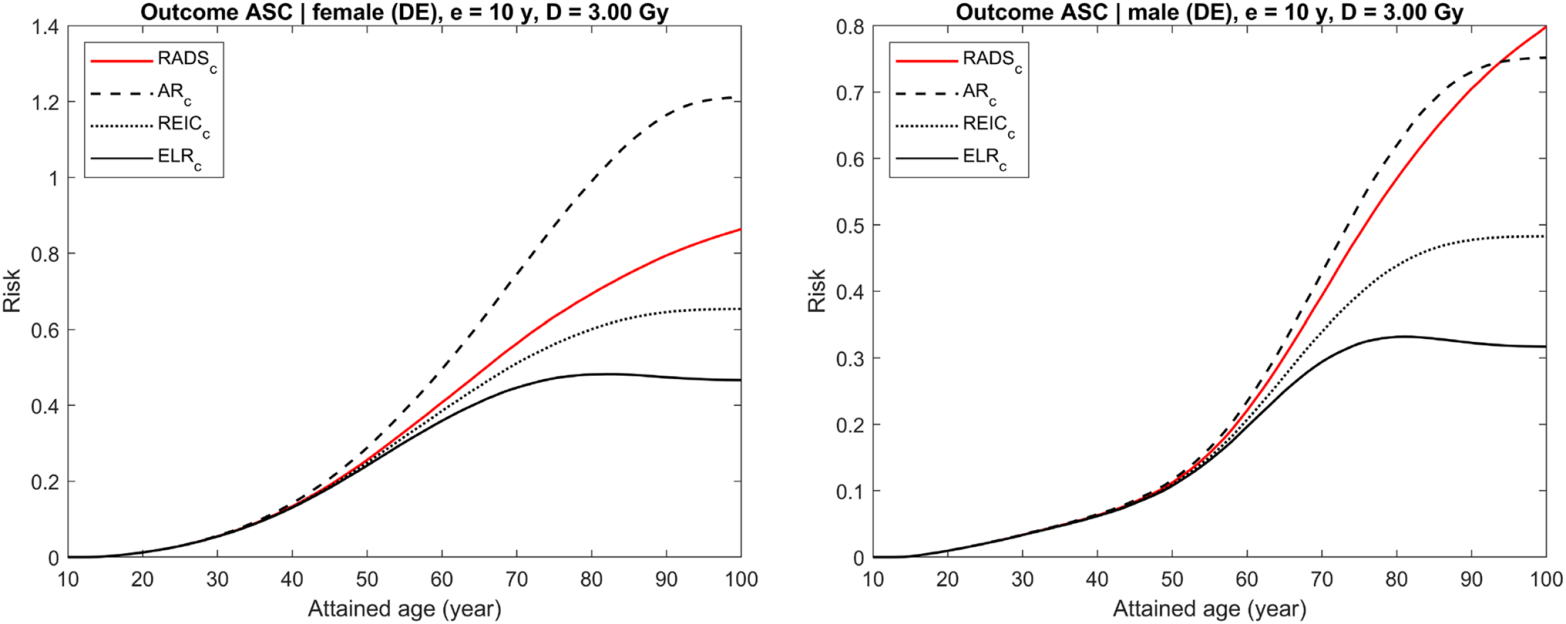
Radiation-attributed risks of all solid cancer incidence for female (left plot) and male (right plot) following exposure at age 10 years to 3 Gy colon dose computed using different risk quantities: AR (black dashed line), REIC (black dotted line), ELR (black solid line), and RADS (red solid line) (colour figure online)

**Fig. 7:**
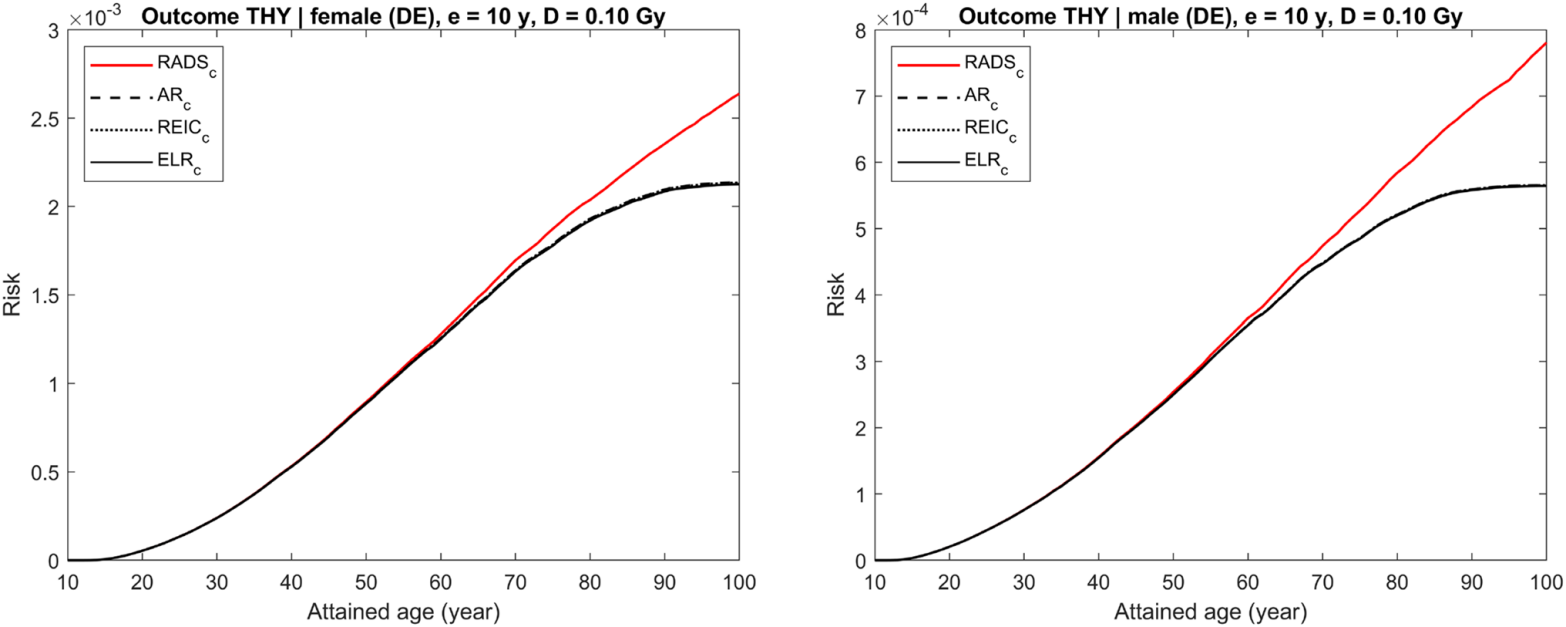
Radiation-attributed risks of thyroid cancer incidence for female (left plot) and male (right plot) following exposure at age 10 years to 0.1 Gy thyroid dose computed using different risk quantities: AR (black dashed line), REIC (black dotted line), ELR (black solid line), and RADS (red solid line) (colour figure online)

**Fig. 8:**
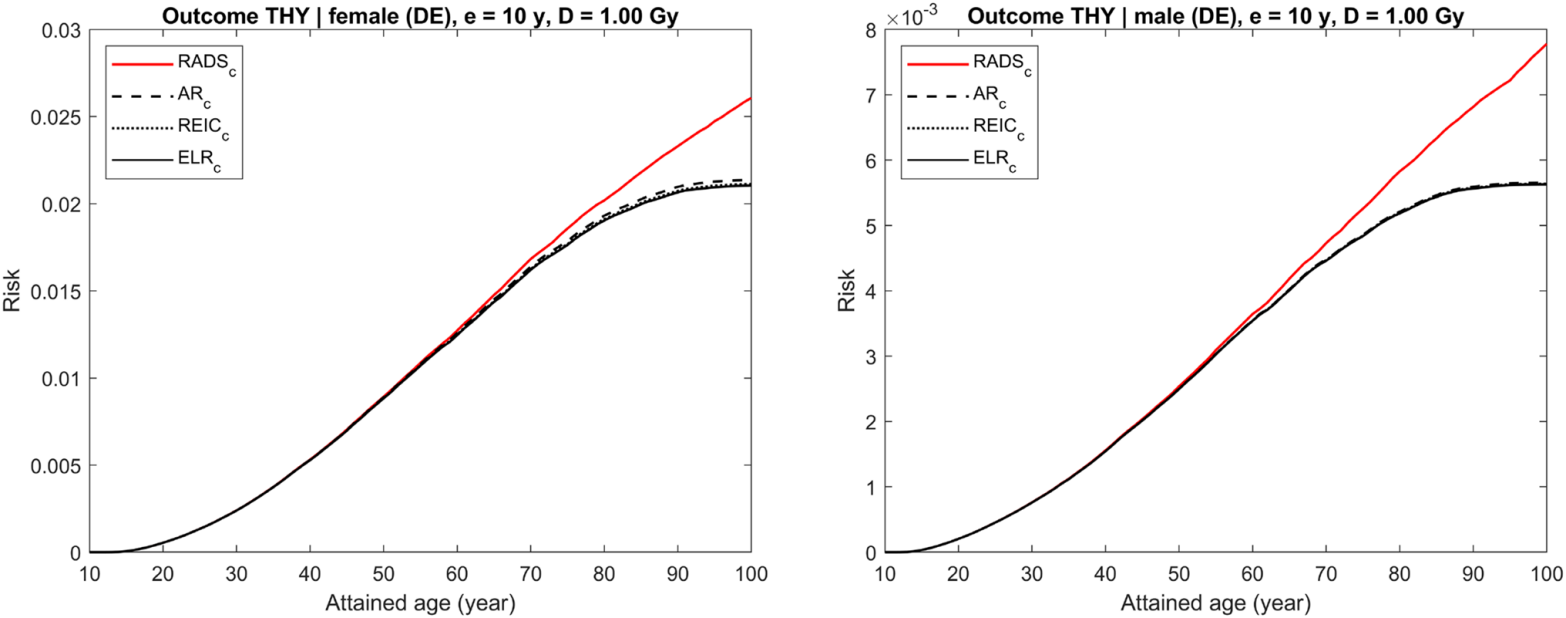
Radiation-attributed risks of thyroid cancer incidence for female (left plot) and male (right plot) following exposure at age 10 years to 1 Gy thyroid dose computed using different risk quantities: AR (black dashed line), REIC (black dotted line), ELR (black solid line), and RADS (red solid line) (colour figure online)

Each figure (Figs. 4, 5, 6, 7, 8) provides results for female (left plot) and male (right plot). The figures represent results for two different outcomes: a composite outcome i.e., all solid cancer incidence (Figs. 4, 5, 6) and a very rare outcome i.e., thyroid cancer (Figs. 7, 8).

For the risk of all solid cancer incidence, Fig. 4 demonstrates the effects of moderate dose exposure to a colon dose of 0.1 Gy. The same estimates are shown in Figs. 5 and 6 for higher values of the colon dose: 1 Gy (Fig. 5) and 3 Gy (Fig. 6). From the figures, it is seen that both AR and REIC overestimate cumulative radiation-attributed risk, represented by excess risk ER. Notably, REIC is always bound between RADS and ER, while AR grows with dose and, at high doses, can result in values exceeding 100% (see Fig. 6, left plot). Another notable effect, which can be seen in Fig. 6, is a reduction of ELR at ages above 75 years. This reduction is due to an inherent deficiency of the ELR definition as a difference of cumulative losses in two populations with different lifetime expectations (Eq. 5), which was pointed out earlier and discussed by Thomas et al. (1992).

The situation changes when the outcome considered is a rare disease, like thyroid cancer, for which the maximum incidence rate in the German population does not exceed $2 \times 10^{ - 4} \;{\text{PY}}^{ - 1}$ (RKI-GEKID 2017). In this case, the effect of the radiation-attributed incidence on the population survival is negligible and, as seen in Figs. 7 and 8, all conventional quantities, AR, REIC, and ELR, are practically coincident for moderate (0.1 Gy, see Fig. 7) and high (1 Gy, see Fig. 8) dose exposures. All these conventional quantities deviate noticeably from RADS at ages older than 60 years, when other, non-radiation attributed, mortality and disease causes significantly affect the remaining survival chances.

## Discussion

Time-integrated risk predictors based on available formalisms (Eqs. 1–7) may be unreliable due to large uncertainties in the future developments of trends in the population-based disease rates, on which they are established. Computations of lifetime or integrated (Eqs. 1–7) risks involve baseline and radiation-attributed rates as well as survival functions. All of these quantities are usually known in retrospective risk assessments, e.g., in retrospective analyses in epidemiological studies, but unknown in prospective studies when integral risk estimates are made at the time of exposure (e.g., in an emergency situation) forward into the future. The extrapolations of survival rates and baseline risks into the future are also problematical if second cancer risk analysis is used in radiotherapy in order to optimise a treatment or as a basis for treatment option choice. Predictors of lifetime risk can be used in radiotherapy treatment planning to optimize a patient specific dose distribution by minimising the corresponding lifetime risk.

In these cases, a problem, inherently bound to risk definitions (Eqs. 1–7), is due to using contemporary population and health statistics data for such risk projections. That is, all estimates of integral risk of this type are conditional on assumption of non-changing secular trends in age- and gender-dependent survival, mortality and incidence data for the target population at the time of risk assessment. Such assumptions for the future developments of secular trends have a degree of implausibility, because survival chances and age-dependent incidence and mortality rates are known to vary with time and can be affected by differences in life-style factors and public health practices throughout the world (see e.g., Forman et al. 2014 and discussions in Walsh et al. 2014). Moreover, uncertainties due to future variations of these quantities are also unknown and cannot be adequately taken into account at the time of risk estimation.

Determination of the survival function $S(t)$ becomes complicated when radiation is applied to treat a cancer. In the case of radiotherapy for cancer, subsequent individual survival chances are significantly different from those for an average member of the general population and need to be considered explicitly using observed relative survival of the cancer patients. The latter depends strongly on the stage of the diagnosed cancer leaving higher chances of survival for patients with diseases diagnosed at early stages and strongly reduced life expectancy for those with cancer diagnosed at later stages, characterised by spread of a tumour and metastases. Correspondingly, estimates of integral risks, based on (Eqs. 1–7), are conditional on survival functions, which can be strongly affected by competing hazardous or beneficial factors, including radiation exposure itself. For example, radiation treatment following breast cancer surgery is known to reduce tumour recurrence and to improve relative survival of the patients (Clarke et al. 2005). Use of the conventional estimates of time-integrated risk (Eqs. 2, 4, 7) creates a so-called ‘RT paradox’, when the highest radiation-attributed lifetime risks of the second primary cancers among patients treated with radiation against cancer are for those whose treatment plan was best leading to improved individual survival chances and extended lifetime.

Survival of radiotherapy patients is not only dependent on tumour stage and cure rates. Also, other factors can have an impact on survival of cancer patients. It is well known that genetic susceptibility underlies some types of cancer (Mack et al. 1995). It is not clear whether this genetic susceptibility would also affect the development of other cancers. There is the possibility of a cancer diathesis, the prospect that, for some reasons related to genetic makeup, a person who developed one cancer has an inherently increased risk of developing another. Such a cancer diathesis would also affect the survival curves of persons who developed a tumour.

Exposure at high doses (say, 1 Gy and beyond) can be associated with detrimental effects resulting in multiple causes of death or diseases (see e.g. Takamori et al. 2017 on comorbidity in the Life Span Study cohort). Risk of radiation detriment to multiple organs affects survival chances, so at high-dose-exposure the basic assumption underlying Eqs. (1–4) becomes invalid (Kellerer et al. 2001), thus resulting in an overestimation of the baseline and attributable risks. The stronger an expected effect of the radiation exposure on survival, the stronger is the degree of overestimation. For compound outcomes, e.g., all malignant diseases, and for high radiation doses, e.g., higher than 1 Gy, conventionally estimated attributable risk (AR/LAR) values exceeding 100% are not unlikely (see e.g. Fig. 6a), thus explicitly demonstrating the implausibility of assumptions underlying the risk calculations and invalidating the corresponding risk estimates. Radiation-attributed competing risks result also in reduction of baseline rates of spontaneous incidence in the exposed population, if compared to the identical non-exposed one (see “Appendix”, Eq. 25).

Conventionally defined and used quantities (Eqs. 2, 4, 7) are approximations to the risk, only valid for limited dose ranges. Their application at higher doses (e.g., exceeding 1 Gy) may result in significant overestimation of the time-integrated (lifetime) radiation-attributable risks. Other methods of risk computation, using ELR (Eq. 5) and ER (Eq. 6), being applicable to any dose range, do not necessarily represent much better approximations to the risk because of the involvement of integrations of failure rates and the requirement of knowledge of survival functions for unexposed and exposed populations as well as time-dependent disease-specific baseline mortality or incidence rates. Therefore, the suggested complementary quantities, baseline and radiation-attributable fractions (Eqs. 11, 12), represent computationally beneficial and practically applicable measures of age-dependent spontaneous and radiation-attributed risks because they require knowledge of only point values of the general survival for the considered population and integrate only radiation-attributable excess rate or baseline rate for the outcome of interest. The attributable fraction (Eqs. 12) is a quantity complementary to time-integrated excess risk (Eq. 6) because it expresses a reduction of chances to survive beyond the age *a* in presence of competing risks, while the ER (Eq. 6) provides an integral probability to die from the cause *c* during the time interval from *e* to *a*.

The application of RADS can be considered to be an important development in attempts to quantify risks to individuals rather than group average risks. This is because the survival curves, based on population all cause and cancer mortality data, are not required in the calculation of RADS, so several sources of uncertainty present in conventionally applied risk measures do not contribute to RADS. However, since RADS does depend on the overall excess hazard, the population cancer rates, which only represent average values for the nationality considered, are still required for the conversion of cohort-specific risk to risk estimates for the target population or sub-group. Therefore, it is important to stress the necessity of avoiding any misunderstandings in the interpretation of the risks calculated with RADS. Risks in terms of RADS, although they can be based on individual doses, cannot completely represent an individual’s cancer risks because there are differences in risk between individuals which go beyond known or measured risk factors (i.e., a frailty variation, see Aalen et al. 2015). An intrinsic frailty variation can influence the levels and uncertainties of the risks in an unknown way, because there is generally no information on important co-factors that influence a particular individual’s cancer risk such as: lifestyle factors (e.g., smoking status and alcohol intake); occupational risk factors; genetic pre-disposition to cancer development; individual radiation sensitivity; and past chemotherapy or radiation medical treatments.

## Conclusions

Conventional, LAR-based, projections of radiation-attributed risk are inappropriate for frequent diseases (i.e., those with a relatively high incidence rate), at high doses, which are common in interventional and therapeutic medical applications, and when survival chances are strongly affected by competing risks (e.g. at older ages or due to a malignant neoplasm and subsequent therapeutic treatment). More general, ELR-based, risk projections are free from dose limitations but are difficult to quantify, especially, when considering effect of spontaneous and attributed competing risks. All conventional quantities are conditional on demographic and health statistical data, for which, future trends are unknown, and on other risk factors affecting survival chances.

For risk projections, where future survival and health statistics are unknown, a quantity, complementary to conventional ones, RADS (Eq. 14) is suggested, which represents risk of radiation detriment only and has the following advantages:

- independence from current and unknown future temporal trends in population survival functions known at the time of estimation; only the estimated radiation-attributed hazard rate is required for this quantity;
- expression of the radiation risk for a specific outcome (disease) that is not sensitive to the effects of radiation on other mortality causes and survival functions;
- aids in avoiding the so-called “RT-paradox” (survival paradox), because the same radiation dose applied for patients with cancer diagnosed at different stages will results in the same radiation risk of a second primary cancer, regardless of the differences in relative survival;
- a higher degree of suitability, than the other risk quantities, for application in risk assessments for other exposed but highly atypical populations (e.g., astronauts) where baseline rates and survival functions pertaining to the general population would be poor approximations (due to distinctly different levels of life-style factors such as smoking and fitness and different levels of cancer screening, post-mission).

It should be noted here that the definition of RADS (Eq. 14), being free from unknown time-dependent epidemiological and demographic data, still involves an integration of model-based outcome-specific excess risk rates $h_{c} (t)$. The latter are typically inferred from epidemiological data defined within certain temporal domains, including ages of exposure and diagnose as well as life span and secular trends of incidence or mortality. Due to this, extrapolation of the model-based excess rates beyond the applicability domain may become a procedure involving unrealistic assumptions; thus, when using RADS, robust excess risk rates $h_{c} (t)$ with highly significant time-dependent model parameters should be preferred.

In medical applications of RADS, the disease incidence should be preferred as an outcome of interest. For example, a radiotherapy patient who is treated for a primary cancer can develop a second cancer. If RADS is used, then it is better to calculate time-integrated risk to develop such a second cancer and not the secondary cancer mortality. The reason for this is, that mortality depends on the future projections of cure rates (of the second cancer) and cure rates are much harder to estimate than incidence rates. The method and equations presented in this study are given for mortality for brevity solely, while the method and the quantities introduced are valid for assessing radiation-attributed risk of cancer incidence, see more on this in “Appendix”.

## Figures and Tables

**Fig. 9:**
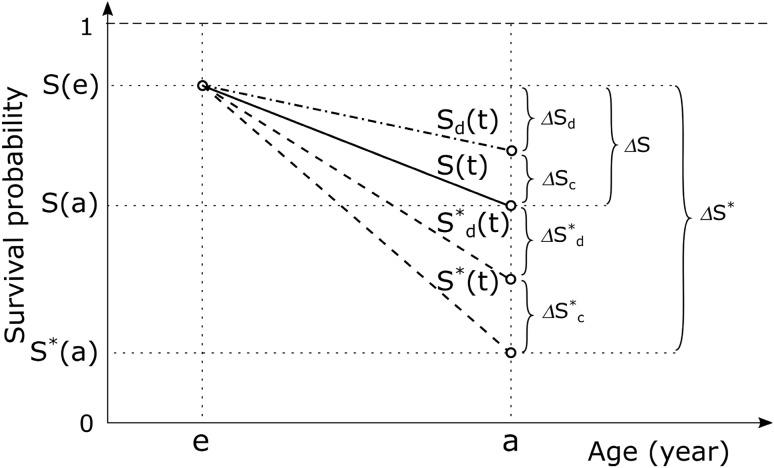
Illustration of survival functions and reduction fractions in the situation of competing radiation-attributed mortality causes. Shown are survival functions and corresponding survival reductions due to: (a) all spontaneous causes but the cause *c*, $S_{d} (t)$ and $\Delta S_{d}$; (b) all spontaneous causes, $S(t)$ and $\Delta S$; (c) all spontaneous and radiation-attributed causes but the cause *c*, $S_{d}^{*} (t)$ and $\Delta S + \Delta S_{d}^{*}$; (d) all spontaneous and radiation-attributed causes, $S^{*} (t)$ and $\Delta S^{*}$

## Appendix

### Main terms of survival statistics and auxiliary equations

In terms of survival statistics (see, e.g., Selvin 1996; Kalbfleisch and Prentice 2002; Kleinbaum and Klein 2012), hazard is a rate of relative change of the survival probability or instantaneous risk:

16 $$\mu (t) = \frac{{{\text{d}}S(t)/{\text{d}}t}}{S(t)},$$

where ${\text{d}}S(t)/S(t)$ is a relative change in survival, i.e., a proportion and can be interpreted as a probability. Correspondingly, by solving the differential equation (Eq. 16), the survival function is related to the hazard as:

17 $$S(t) = \exp \left( { - \mathop \int \limits_{0}^{t} \mu (u){\text{d}}u} \right) .$$

Consider estimates of conditional survival, $S\left( {a |e} \right) \equiv S(a)/S(e)$, i.e., a probability to survive to age *a* for members of an unexposed population alive at age *e*:

18 $$S\left( {a|e} \right) = \exp \left( { - \mathop \int \limits_{e}^{a} \mu (u){\text{d}}u} \right) = \exp \left( { - \mathop \int \limits_{e}^{a} \mathop \sum \limits_{i} \mu_{i} (u) {\text{d}}u} \right) = \mathop \prod \limits_{i} \exp \left. {\left( { - M_{i} (a|e)} \right.} \right) = \mathop \prod \limits_{i} S_{i} (a|e),$$

where $\mu_{i} (u)$ is the mortality rate and $M_{i} \left( {a |e} \right) = \int_{e}^{a} {\mu_{i} (u ) {\text{d}}u}$ is the cumulative mortality of the *i*th cause, integrated from age *e* to age *a*. As seen from Eq. (18), the ‘all-cause’ survival is expressed as a product of partial conditional survival factors $S_{i} (a|e)$ reflecting the effects of cause-specific hazards.

Factorization (Eq. 18) opens possibilities for alternative formulations (Eqs. 11, 12, 14) of radiation-attributed excess risk introduced in the paper.

### Survival reduction in the presence of competing radiation-attributed causes

Now, consider radiation-attributed and spontaneous survival reductions for the cause of interest *c* in the more complicated situation where radiation affects not only the main cause of interest but also other causes, which appear competitive to the main cause of interest. Figure 9 illustrates the corresponding survival curves and survival reduction fractions for this situation. Similar to Fig. 1 in the main text, shown in the figure are probabilities for a member of unexposed population to survive all mortality causes, $S(t)$, and all-but-cause-*c*, $S_{d} (t)$, and probabilities for a member of exposed population to survive all mortality causes, $S^{*} (t)$, and all-but-cause-*c*, $S_{d}^{*} (t)$. Correspondingly, survival reductions at age *a* are: (a) due to all mortality causes in the unexposed population, $\Delta S$; (b) due to all causes in the exposed population, $\Delta S^{*}$; (c) due to the cause *c* in the unexposed population, $\Delta S_{c}$; (d) due to all-but-cause-*c* in the unexposed population, $\Delta S_{d}$; (e) due to cause *c* in the exposed population, $\Delta S_{c}^{*}$; (f) due to all-but-cause-*c* in the exposed population, $\Delta S_{d}^{*}$.

And again, as applied in the main manuscript, all quantities are conditional on survival until age *e* (i.e. $S(e) \equiv 1$) and, for brevity, the simplified notations (see Table 1) are used in the equations below. Under these assumptions, the survival function for the exposed population can be factorised and expressed by applying other survival functions as follows (see Fig. 9 and Table 1 for the notations):

19 $$\begin{aligned} S^{*} (a) & = \exp \left( { - M_{d} (a)} \right)\exp \left( { - M_{c} (a)} \right)\exp \left( { - H_{d} (a)} \right)\exp \left( { - H_{c} (a)} \right) \\ & = S_{d} (a)\exp \left( { - M_{c} (a)} \right)\exp \left( { - H_{d} (a)} \right)\exp \left( { - H_{c} (a)} \right) \\ & = S(a) \exp \left( { - H_{d} (a)} \right)\exp \left( { - H_{c} (a)} \right) \\ & = S_{d}^{*} (a) \exp \left( { - H_{c} (a)} \right). \\ \end{aligned}$$

Correspondingly, the attributable fraction representing the share of population alive at age *e*, but not surviving to age *a*, due to the radiation-attributed cause *c*:

20 $${\text{AF}}_{c} (a) = \Delta S_{c}^{*} = S_{d}^{*} (a) - S^{*} (a) = S(a)\exp \left( { - H_{d} (a)} \right)(1 - \exp ( - H_{c} (a))),$$

and the spontaneous cause *c*:

21 $${\text{BF}}_{c} (a) = \Delta S_{c} = S_{d} (a) - S(a) = S(a)(\exp (M_{c} (a)) - 1).$$

Similarly, a representation of RADS in the presence of radiation-attributed competing risks is as follows:

22 $${\text{RADS(}}a )= \exp ( - H_{d} (a))(1 - \exp ( - H_{c} (a))).$$

### Time-integrated excess risk in the presence of competing radiation-attributed causes

The attributed survival fractions indicate chances to survive beyond the certain time point. This make them different from the conventional integrated risk quantities (Eqs. 1–7), which represent integral losses due to specific cause during a given period. Still, the factorisation of survival functions (Eq. 19) can be helpful in gaining a better understanding of the assumptions and limitations inherent to the conventional LAR and AR quantities.

Consider, excess risk, ER, due to radiation-attributed cause *c* in the situation when other mortality causes are also affected by the radiation exposure. From the general definition of ER (Eq. 6):

23 $${\text{ER}}_{c} (a) = \mathop \int \limits_{e}^{a} \left[ {\mu_{c}^{*} (u) S^{*} (u) - \mu_{c} (u) S(u)} \right]{\text{d}}u,$$

and factorisation (19), using notations from Table 1:

24 $${\text{ER}}_{c} (a) = \mathop \int \limits_{e}^{a} S(u) \mu_{c} (u)\left( {\left( {\frac{{h_{c} (u)}}{{\mu_{c} (u)}} + 1} \right)\exp \left( { - H_{d} (u)} \right)\exp \left( { - H_{c} (u)} \right) - 1} \right){\text{d}}u.$$

From the above equation it can be seen that, for low-dose exposures, when radiation attributable excess rates are low, $H_{d} (t) \ll 1$ and $H_{c} (t) \ll 1$, so the terms $\exp ( - H_{d} (t)) \approx 1$ and $\exp ( - H_{c} (t)) \approx 1$; then the ELR converges to the well-known definition of attributable risk AR (i.e., LAR, if the upper integration limit represents age at end of lifetime).

Equation (24) provides an accurate method of excess risk calculation taking into account effect of radiation-attributed competing risks.

Competing radiation-attributed risks of other causes than the cause *c* effect also the baseline (spontaneous) risk of the cause *c* in the exposed population:

25 $$\begin{aligned} {\text{BR}}_{c} (a) & = \mathop \int \limits_{e}^{a} \mu_{c} (u)S^{*} (u){\text{d}}u \\ & = \mathop \int \limits_{e}^{a} \mu_{c} (u)\exp \left( { - H_{c} (u) - H_{d} (u)} \right)S(u){\text{d}}u \\ & = \mathop \int \limits_{e}^{a} \mu_{c}^{\prime } (u)S(u){\text{d}}u, \\ \end{aligned}$$

where $\mu_{c}^{\prime } (u) = \mu_{c} (u)\exp ( - H_{c} (u) - H_{d} (u))$ is the baseline mortality rate in the exposed population reduced by the effect of competing radiation-attributed mortality rates.

### Disease incidence and disease-free survival

In the main paper, all equations are given for mortality rates and survival functions represent chances to survive beyond the certain limit. If the outcomes of interest are not fatalities but disease occurrences, then all the major results and conclusions presented in the paper remain valid provided that the survival functions are redefined now to express the probability of being alive and disease-free at certain time. Technically, this means that all baseline mortality rates need to be replaced with corresponding incidence rates

26 $$\mu (t) \to \mu_{0} (t) + \lambda (t), \mu_{c} (t) \to \lambda_{c} (t), \mu_{d} (t) \to \lambda_{d} (t),$$

where $\mu_{0} (t)$ is the mortality rate from instant, non-disease specific, causes, such as car accidents, and the excess rates $h(t), h_{c} (t), h_{d} (t)$ now represent excess incidence rates and the survival functions are correspondingly redefined as the disease-free survival functions:

27 $$\begin{aligned} S^{*} (a) & = \exp ( - M_{0} (a))\exp ( - \varLambda_{d} (a))\exp ( - \varLambda_{c} (a))\exp ( - H_{d} (a))\exp ( - H_{c} (a)) \\ & = S_{d} (a)\exp ( - \varLambda_{c} (a))\exp ( - H_{d} (a))\exp ( - H_{c} (a)) \\ & = S(a) \exp ( - H_{d} (a))\exp ( - H_{c} (a)) \\ & = S_{d}^{*} (a) \exp ( - H_{c} (a)), \\ \end{aligned}$$

where $M_{0} (t) = \int_{e}^{a} {\mu_{0} (u ) {\text{d}}u}$ is the cumulated mortality rate from the instant, not related to diseases, causes; and $\varLambda_{c} (t)$, $\varLambda_{d} (t)$, $H_{c} (t)$, and $H_{d} (t)$ are the respective cumulated baseline and excess incidence rates.

Then, all of the main quantities derived in the present paper remain applicable to attributed and spontaneous risks and disease-free-survival chances for developing a specific disease after being exposed to radiation.

In practical situation, it is common that the general population survival curve, $S_{\text{LT}} (t)$, as carefully defined in officially published statistical life-tables, is preferably applied in calculations. Then, the conversion to the disease-free survival, $S(t)$, can be done by taking into account difference between all-cause disease incidence and mortality rates:

28 $$S(t) = S_{\text{LT}} (t)\exp \left( { - \mathop \int \limits_{e}^{t} (\lambda (u) - \mu (u)){\text{d}}u} \right).$$

## Footnotes

1 For conceptual ease, failure rates considered here are represented by mortality rates only. Consideration of radiation risks of being diseased and of corresponding incidence rates requires use of disease-free survival chances and can be achieved by a re-definition of the hazard rates. See more on this in the “Appendix”.

2 The number of failure cases (here, deaths) per person-year abbreviated as PY.

3 Here we do not consider the problem of transfer of the model risk to the target population. Type of the risk transfer, additive or multiplicative, should be differentiated from a type of a phenomenological model used to parameterise radiation risk. In the current paper, the excess rate $h_{c}$ is the pure model-based estimate by whatever, EAR or ERR, type of a phenomenological model. A description of a pertinent risk transfer technique can be found elsewhere (see Jacob et al. 2013; Ulanowski et al. 2016).

4 Recall that the notations, introduced in Table 1, are already conditional on survival to age of exposure *e*, thus $S(e) \equiv 1$.
